# Variant graph craft (VGC): a comprehensive tool for analyzing genetic variation and identifying disease-causing variants

**DOI:** 10.1186/s12859-024-05875-7

**Published:** 2024-09-03

**Authors:** Jennifer Li, Andy Yang, Benedito A. Carneiro, Ece D. Gamsiz Uzun, Lauren Massingham, Alper Uzun

**Affiliations:** 1 Department of Computer Science, Brown University, Providence, RI 02912 USA; 2 Department of Chemistry, Brown University, Providence, RI 02912 USA; 3 Lifespan Cancer Institute, Providence, RI 02912 USA; 4 Legorreta Cancer Center, Brown University, Providence, RI 02912 USA; 5 Department of Pathology and Laboratory Medicine, Rhode Island Hospital, Providence, RI 02912 USA; 6 Center for Computational Molecular Biology, Brown University, Providence, RI 02912 USA; 7 Department of Pathology and Laboratory Medicine, Alpert Medical School, Brown University, Providence, RI 02912 USA; 8 Department of Pediatrics, Division of Genetics, Warren Alpert Medical School, Brown University, Providence, RI 02912 USA; 9 Center for Clinical Cancer Informatics and Data Science (CCIDS), Brown/Lifespan, Providence, RI 02912 USA; 10 Department of Pediatrics, Warren Alpert Medical School, Brown University, Providence, RI 02912 USA

**Keywords:** Genomic variation, Variant call format (VCF), Variant graph craft (VGC), Visualization, Genomic data analysis, Genotype information, Gene function, Pathogenic variants, Data security, User-friendly interface

## Abstract

**Background:**

The variant call format (VCF) file is a structured and comprehensive text file crucial for researchers and clinicians in interpreting and understanding genomic variation data. It contains essential information about variant positions in the genome, along with alleles, genotype calls, and quality scores. Analyzing and visualizing these files, however, poses significant challenges due to the need for diverse resources and robust features for in-depth exploration.

**Results:**

To address these challenges, we introduce variant graph craft (VGC), a VCF file visualization and analysis tool. VGC offers a wide range of features for exploring genetic variations, including extraction of variant data, intuitive visualization, and graphical representation of samples with genotype information. VGC is designed primarily for the analysis of patient cohorts, but it can also be adapted for use with individual probands or families. It integrates seamlessly with external resources, providing insights into gene function and variant frequencies in sample data. VGC includes gene function and pathway information from Molecular Signatures Database (MSigDB) for GO terms, KEGG, Biocarta, Pathway Interaction Database, and Reactome. Additionally, it dynamically links to gnomAD for variant information and incorporates ClinVar data for pathogenic variant information. VGC supports the Human Genome Assembly Hg37 and Hg38, ensuring compatibility with a wide range of data sets, and accommodates various approaches to exploring genetic variation data. It can be tailored to specific user needs with optional phenotype input data.

**Conclusions:**

In summary, VGC provides a comprehensive set of features tailored to researchers working with genomic variation data. Its intuitive interface, rapid filtering capabilities, and the flexibility to perform queries using custom groups make it an effective tool in identifying variants potentially associated with diseases. VGC operates locally, ensuring data security and privacy by eliminating the need for cloud-based VCF uploads, making it a secure and user-friendly tool. It is freely available at https://github.com/alperuzun/VGC.

## Introduction

In recent years, advancements in genome sequencing technologies have enabled researchers to generate vast amounts of genomics data. However, with this flood of information comes the need for tools that can analyze and visualize this data effectively. One of the key challenges in analyzing genetic data is dealing with the complexity and the size of variant data stored in VCF files. These files contain information about genetic variations, including single nucleotide polymorphisms (SNPs), insertions, deletions, and structural variations. Analyzing VCF files is a complex task that necessitates several steps, including indexing, filtering, extracting, visualization, and detailed analysis of genetic variations, preferably with annotations. The conventional approach to VCF file visualization predominantly relies on command-line tools, posing a significant challenge for those not well-versed in terminal-based operations.

While existing tools offer summaries and some level of interactivity, they face notable challenges, particularly in scalability and user-friendliness. One of the primary issues is scalability; handling large datasets can be daunting due to performance bottlenecks and inefficient data processing. This scalability challenge stems from the inherent complexity and size of genomic data, which requires robust and efficient tools to manage effectively [1]. Current tools such as vcflib, bio-vcf, cyvcf2, hts-nim, slivar and re-Searcher have been developed to provide solutions for processing VCF files, aiming to mitigate the scalability issue by optimizing for large datasets [2, 3]. Another limitation of these tools is the lack of or limited interactivity, as many of them do not provide dynamic and interactive environments for exploring variant data. This can make it difficult for researchers to fully understand and analyze the data and explore potential associations between genetic variants and phenotypes. In addition, some of the existing VCF file visualizing tools can be confusing to use and may require significant expertise to operate effectively. Some tools have too many dependencies based on the origin of the programming language and new updates may crash the program, which can add to the complexity of using these tools. Furthermore, compatibility issues may arise due to the different VCF file formats used by different tools, which can make it difficult to compare results between different tools.

To address these challenges and limitations, several user-friendly VCF file visualization and analysis tools have been developed that offer a wide range of features for visualizing genetic variations and exporting filtered data. In the field of genomic research, there are several well-known bioinformatics tools that significantly enhance data analysis and visualization capabilities. These include IGV (Integrative Genomics Viewer), which offers an interactive platform for genomic datasets visualization [4]; VCF-Server, tailored for managing and querying VCF files [5]; VCF. Filter, allowing for the intricate filtering of VCF files [6]; and BrowseVCF, providing a user-friendly interface for VCF file exploration [7]. Additionally, GEMINI (Genome Exploration and Mining INteractive Interface) focuses on the integrative analysis and variant prioritization within VCF files [8]. VCF-Miner is a standalone, GUI-based tool for mining and filtering VCF file variants, using a MongoDB engine to identify relevant variants in various organisms [9]. VCFtools is a comprehensive package for manipulating and interpreting VCF files, including data comparison, summarization, and statistical analysis [10]. Visualization of Variants (VIVA) is designed for the intuitive visualization and analysis of genomic variants, facilitating complex data interpretation through a graphical interface [11]. Together, these tools form a robust suite for genomic data management, analysis, and visualization, catering to a variety of research needs in the genomics field. However, despite the improvements made, there is still room for further enhancements to improve scalability, customizability, interactivity, complexity, and compatibility. To overcome these limitations, we have developed Variant Graph Craft (VGC), a VCF analysis and visualization tool designed to extract and visualize variant data from VCF files with multiple customizable options. VGC designed primarily for analyzing patient cohorts. However, VGC can also be adapted for the analysis of individual probands or families, providing flexibility for various research and clinical scenarios.

In addition to the tools for VCF visualization and analysis, the field of rare disease analysis benefits from numerous VCF annotation, filtering, and prioritization tools that integrate patient phenotype information. According to a comprehensive evaluation by Yuan et al. over 20 such tools, including both open-source and commercial options, have been developed to enhance the identification of disease-causing genes in patients with Mendelian disorders [12]. Tools like LIRICAL, AMELIE, and Exomiser, which use Human Phenotype Ontology (HPO) terms in conjunction with VCF files, have shown superior performance in accurately prioritizing candidate genes compared to those relying solely on phenotypic data [13–16].

VGC adeptly addresses several challenges associated with the analysis and visualization of genetic variation data from VCF files through a multitude of innovative features. It provides a solid platform for comprehensive variant data extraction and visualization, enabling users to efficiently browse through genetic variations with details on variant positions, alleles, genotype calls, and quality scores. By transforming complex genomic data into interactive graphical representations, VGC facilitates easy identification of patterns across samples, enhancing the understanding of genetic landscapes. The integration of information from publicly available databases such as MSigDB, KEGG, Biocarta, Pathway Interaction Database (PID), Reactome, gnomAD, and ClinVar enriches the analysis with valuable insights into gene functions, variant frequencies, and pathogenic variants. Operating locally, VGC ensures the privacy and security of sensitive genomic data, a critical feature that sidesteps the need for cloud uploads and thus addresses significant privacy concerns. Its compatibility with the Human Genome Assembly Hg37 and Hg38 ensures that VGC is adaptable and applicable to a wide array of genomic studies. Furthermore, the tool's ability to incorporate optional phenotype input data allows for customized analysis tailored to specific research questions or clinical contexts, thereby facilitating deeper investigations into genotype–phenotype relationships. Through these features, VGC overcomes scalability, interactivity, complexity, and data security challenges, establishing itself as a valuable resource for researchers and clinicians working in genomic variation analysis.

## Implementation

**Fig. 1:**
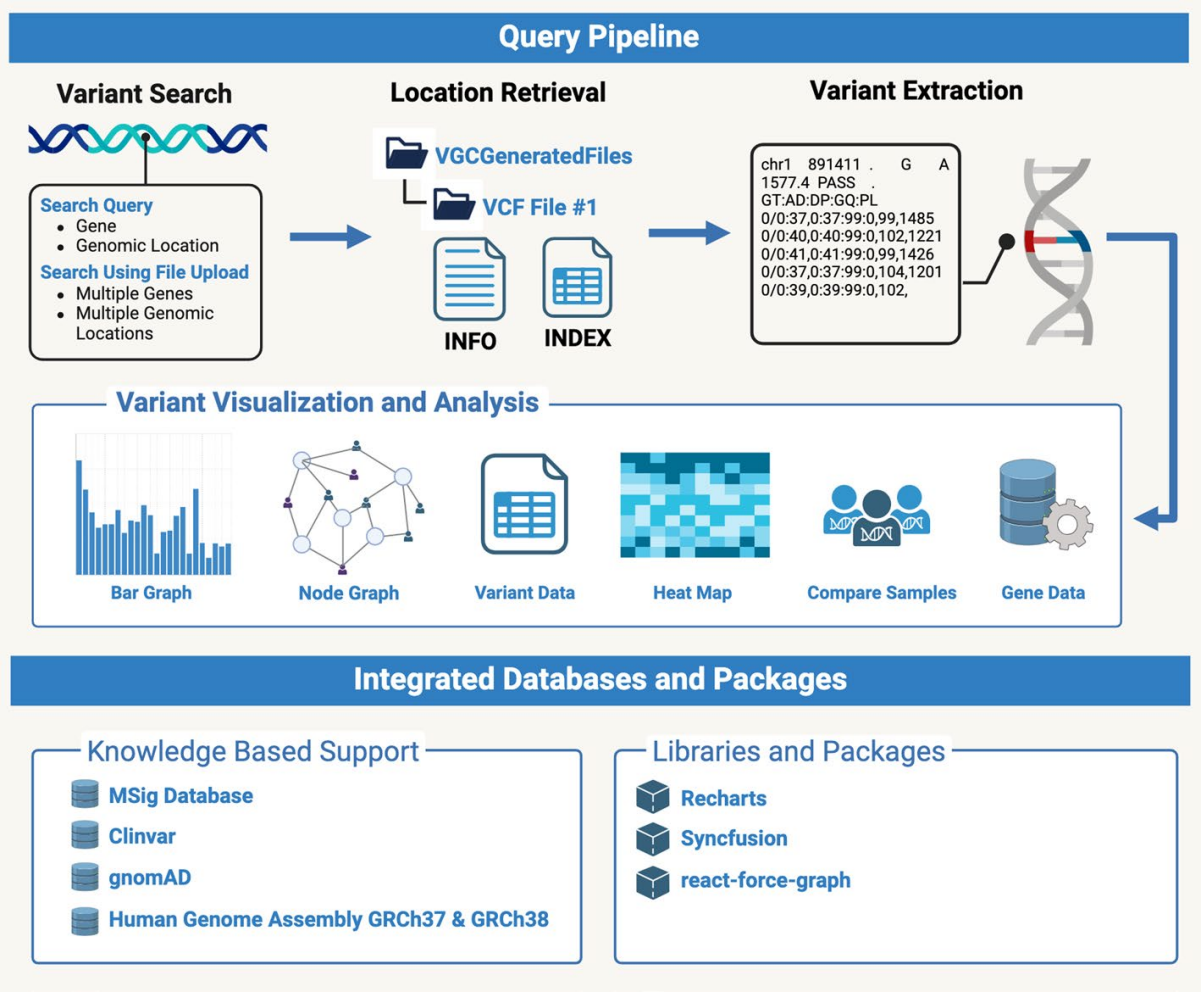
Design and integration of VGC. The query pipeline of VGC offers four distinct search options, as well as knowledge-based support with visualization and analysis. Within a given VCF file, users may choose to query single gene names or genomic locations as well as multiple genes or genomic locations simultaneously via file upload options. Relevant information pertaining to the queried variants is retrieved from stored files, thus allowing for efficient variant extraction from the uploaded VCF. The identified variations may then be displayed using interactive graphics, such as histograms, node graphs, spreadsheets, heat maps, sample comparisons, and gene data visualization. The pipeline is supported by several integrated databases and packages, allowing for rich analyses and visualizations

VGC is a tool designed for analyzing variant data and visualizing VCF files. It utilizes a range of technologies and libraries to offer a user-friendly experience (Fig. 1).

### Programming languages, applications and libraries

VGC is a desktop application created using a JavaScript frontend and Java backend. The application is currently built using webpack [17] module bundler version 5.86.0, and packed for iOS, Windows, and Linux using electron-forge [18]. Communication between the frontend and backend of VGC is handled by the Axios HTTP library [19]. VGC is currently packaged using Electron for deployment, which allows the tool to be easily installed and run on a wide range of platforms and operating systems [20].

UI components are created using the React framework [21] version 18.2.0, and styled using Tailwind CSS [22]. To generate highly interactive and dynamic graphics for data visualization, the application utilizes a range of libraries, including Syncfusion [23], react-force-graph [24], and Recharts [25]. These libraries provide a range of tools and functionalities for the visualization and analysis of complex data sets.

### Integration of publicly available databases

VGC draws from a range of public databases, including MSig Database for GO terms, as well as KEGG, Biocarta, PID, and Reactome [26–30]. By leveraging these powerful databases, VGC is able to provide users with rich and detailed information about the genetic pathways and functions associated with their variant data, allowing for deeper insights and a greater understanding of the underlying biology. VGC also includes a dynamic link to gnomAD for variant information, allowing users to easily access and explore genetic variation data from this well-known database [31]. Additionally, the tool includes ClinVar data for pathogenic variant information, providing users with different visualization options for identifying and understanding potentially harmful genetic mutations [32]. VGC supports the Human Genome Assemblies GRCh37 and GRCh38, ensuring compatibility with a wide range of data sets. The tool provides a range of options for exploring genetic variation, and can be tailored to the specific needs of the user by using optional phenotype input data.

#### Dynamic link to gnomAD for variant information

The dynamic link feature of VGC to gnomAD, a widely-used database for variant information provides users with a seamless connection to gnomAD, allowing them to access up-to-date and comprehensive variant data. The decision to implement a dynamic link specifically to gnomAD, as opposed to other databases, stems from its unique role as an aggregation database of genetic variation. This distinctive feature consolidates variant information from a variety of sources, providing a comprehensive resource. By establishing this dynamic link, VGC ensures that users have access to the latest information on variant frequencies and population-specific data. This integration enhances the accuracy and reliability of variant interpretation, empowering researchers to make informed decisions based on the most current genomic data available.

#### Incorporation of ClinVar data for pathogenic variant information

Inclusion of ClinVar data within VGC provides information on pathogenic variants and their clinical significance. By incorporating ClinVar data, VGC enables users to assess the potential pathogenicity of identified variants. Users can access curated information on variants that have been associated with specific diseases or conditions. This integration aids in variant prioritization, helping users focus on variants that may have clinical implications and guiding further investigation.

#### Compatibility with human genome assemblies GRCh37 and GRCh38

VGC is designed to work seamlessly with these widely-used genome assemblies, ensuring compatibility with a broad range of datasets. By supporting both GRCh37 and GRCh38, VGC enables users to analyze genomic variation data generated using different platforms and datasets aligned to these assemblies. This compatibility enhances the versatility and applicability of VGC, making it a valuable tool for a wide range of genomics studies and research projects.

### User input and preprocessing

**Fig. 2:**
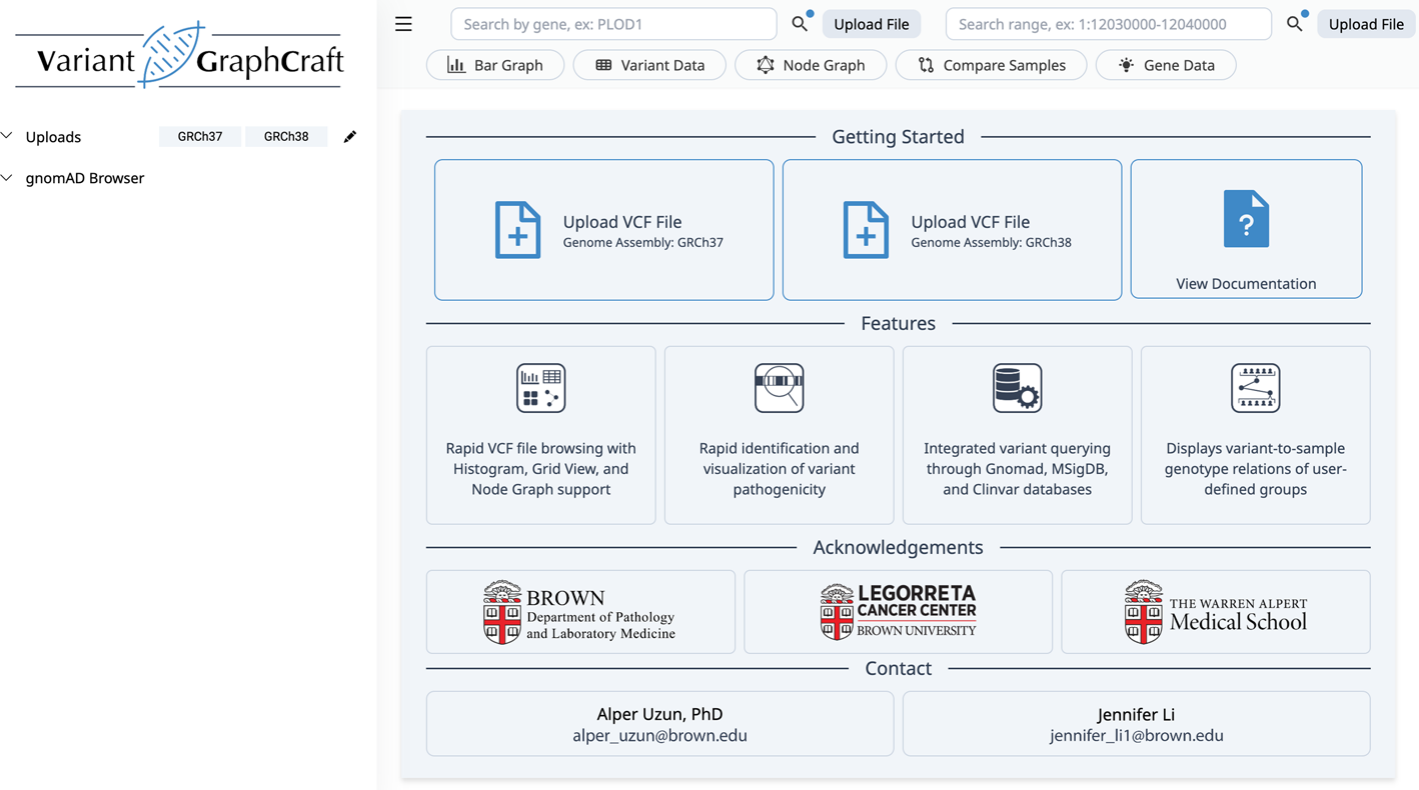
VGC user interface on startup. Users may begin an analysis by selecting a genome assembly (GRCh37 or GRCh38) and uploading the respective VCF file

Upon opening, VGC displays a “welcome” page, allowing users to begin analyses for genome assemblies GRCh37 or GRCh38 (Fig. 2). For a given analysis, users may input two files: (1) a required VCF file, and (2) a supplemental and optional phenotype file specifying sample groupings.

#### Extraction and indexing of VCF

When a new VCF file is uploaded to the program, VGC processes it to extract pertinent information, which is then stored in the user's file system. A new directory named “VGCGeneratedFiles” is created in the user's home directory, along with a corresponding directory that follows a specific naming scheme.

For each VCF file processed, a directory named “VGC_<filename>” is created. Inside these directories, two text files, named info_<filename> and index_<filename>, store important data. The info_<filename> file holds overall file information, such as the VCF file version, total number of samples, total number of chromosomes, number of variants, the header line, and a list of chromosomes in the file. The index_<filename> file contains chromosome-specific information. This indexing by VGC enhances response times for future queries. For each chromosome in the VCF file, the following details are listed in the index file: starting and ending lines, starting and ending positions, number of variants marked as “PASS,” and the count of pathogenic variants for that chromosome.

#### Customization to suit individual user requirements by incorporating optional phenotype input data

VGC allows users to incorporate additional phenotype information, aligning the analysis with specific research questions or clinical contexts. By incorporating phenotype input data, VGC enables users to explore genetic variations in the context of specific phenotypic traits, enhancing the understanding of genotype–phenotype relationships. This customization feature makes VGC adaptable to various research and clinical scenarios, ensuring that users can leverage the tool to its full potential in their specific domain of interest.

### User queries and visualization

#### Query options

Users have the flexibility to search for specific genes or defined genomic ranges within the VCF file, enabling focused analysis of variants. When searching by gene, all variants corresponding to that gene within the VCF file are visualized. Alternatively, users can specify a genomic range, extracting and visualizing variants within the defined interval.

The variant extraction process utilizes the information stored in the index_<filename> file, which, as described earlier, provides the starting and ending lines of chromosomes within the VCF file. Depending on the user's selection of GRCh37 or GRCh38 as the reference genome assembly, the system accurately retrieves the relevant variants. Additionally, users can streamline their analysis by uploading a file containing multiple genes or genomic ranges, facilitating simultaneous querying of multiple genes or ranges. Variants associated with each queried gene or range are then extracted and visualized.

#### Visualization options

VGC offers a diverse range of visualization options tailored to meet various analytical needs.

When a VCF file is initially uploaded, a default bar graph view will display all variants by chromosome present in the file, with each bar corresponding to the number of variants within a specific chromosome. Users can navigate through viewing history using forward and backward arrows. Hovering over a bar reveals details indicating the number of variants displayed as well as the corresponding genomic range. Clicking on a bar enables zoom functionality for a closer examination of variants within the selected data.

Variant data may also be presented in a structured table format, enhancing accessibility and ease of analysis. User may choose to filter, sort, export, or other manipulate data in a spreadsheet-like display.

For analysis of case–control studies, sample groupings, or sample genotypes, VGC provides a node graph visualization option. Users may toggle between 2 and 3D views, facilitating interactive exploration of variant relationships. Moreover, the tool provides Fisher’s Exact Test data for each variant relative to sample groups. The test assesses differences in variant abundance between designated groups (e.g., cases vs. controls) through Monte Carlo simulation. By analyzing a 2 × 3 matrix with default simulations (n = 2000), potential associations between variants and sample groups can be discerned, aiding in phenotype-genotype analyses.

### Secure and private local environment for data analysis

VGC is designed to run on the local machine or servers, ensuring that users can work with their genomic data in a secure and confidential setting. By avoiding the need to upload VCF files to the cloud, VGC protects sensitive genomic data and addresses privacy concerns. This local deployment approach instills a sense of reassurance in users, as they can confidently maintain control over their data, ensuring it stays within their organization's infrastructure. VGC requires Java version 1.8 or higher to run and is compatible with Windows, Mac, and Linux, offering flexibility for users across different platforms.

## Results

**Fig. 3:**
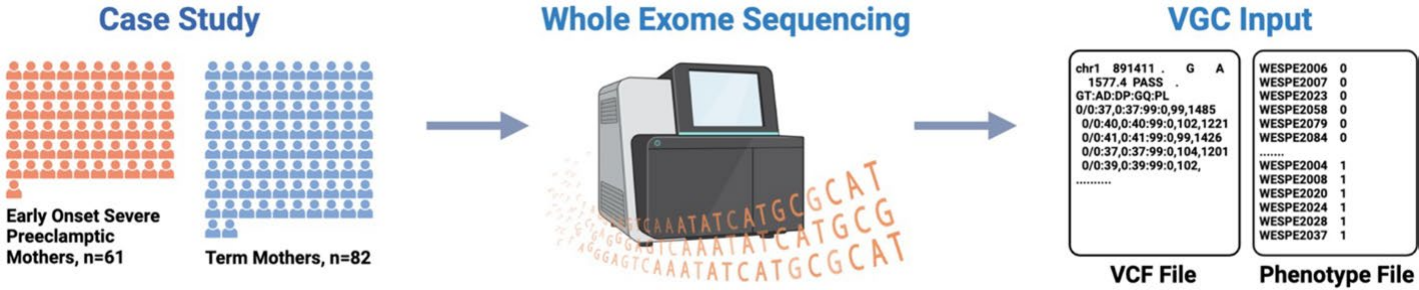
Schematic overview of case–control study to VGC input. To illustrate VGC's capabilities, we present a case study of early onset severe preeclamptic mothers (n = 61) and term mothers (n = 82). Whole exome sequencing of the described case–control samples and subsequent variant calling allowed for the creation of (1) a VCF file and (2) a customized phenotype file as VGC input

VGC features advanced visualization tools for VCF files. Demonstrating VGC's capabilities, we present an example using whole exome sequencing data from preeclamptic patients and term mothers (Fig. 3). The dataset includes 143 samples: 61 early onset severe preeclamptic cases and 82 term mother controls [33]. Through VGC, we offer a detailed analysis of this dataset, emphasizing major trends, statistical findings, and key outcomes aligned with our research goals. The insights gleaned from this study significantly enhance our understanding of variants associated with preeclampsia and offer valuable information for future research and practical applications.

### Comprehensive variant data extraction and visualization

**Fig. 4:**
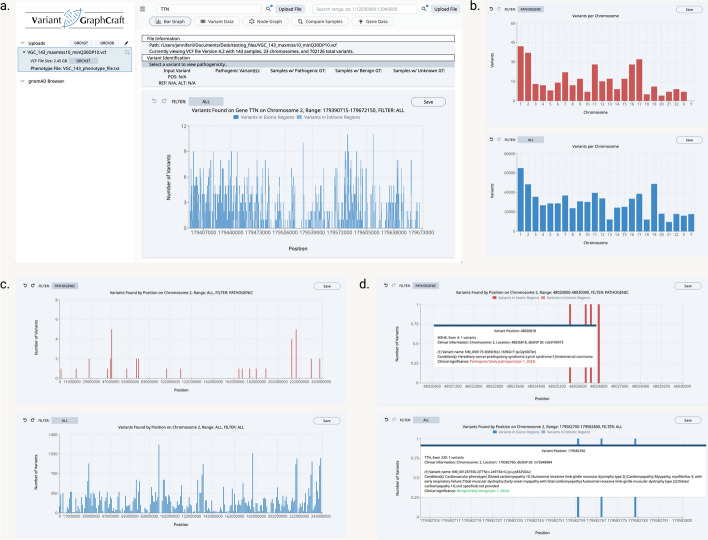
Histogram-based variant browsing with VGC. **a** The VGC user interface upon query of TTN, a gene found to contain pathogenic variants in the uploaded file. **b** Variants per chromosome, non-filtered [top] vs. filtered by pathogenicity [bottom]. **c** Partially magnified view of variants in CHR 1 for non-filtered [top] vs. filtered by pathogenicity [bottom]. **d** A detailed tooltip containing ClinVar-based information appears on hover when magnified to the single-position increment

VGC excels in variant browsing, offering features that enable effective exploration and analysis of genetic variations. It efficiently retrieves crucial data such as variant positions, alleles, genotype calls, and quality scores, offering a comprehensive and structured view of genomic variations for researchers and clinicians. For example, we demonstrate the visualization of variants in TTN, a gene with pathogenic, nominally significant variants identified in univariate analysis (Fig. 4). TTN variants are displayed in a histogram, sorted by variant position. Variants in intronic and exonic regions are differentiated by color (Fig. 4a). Users have the option to filter variants by categories such as “ALL,” “PASS,” or “Pathogenic”. VGC’s visualization capabilities extend beyond basic displays, offering sophisticated graphical representations that deepen understanding of variant data (Fig. 4b–d). Its intuitive and interactive visualizations allow users to discern patterns, connections, and insights within the genomic variations. In these analyses, such as when visualizing variants of the TTN gene, users have the option to save the variant list with all existing features from the VCF file in four different file formats (.xlsx,.xls,.csv,.pdf). This functionality allows users to retain the gene of interest for later examination and facilitates the transfer of these files for further analysis. Additionally, after the initial presentation of the VCF file, subsequent sessions will benefit from quicker access since the file will have been indexed, enabling more efficient and rapid visualization for repeated use of the same files.

### Graph representation of samples and genotype data

**Fig. 5:**
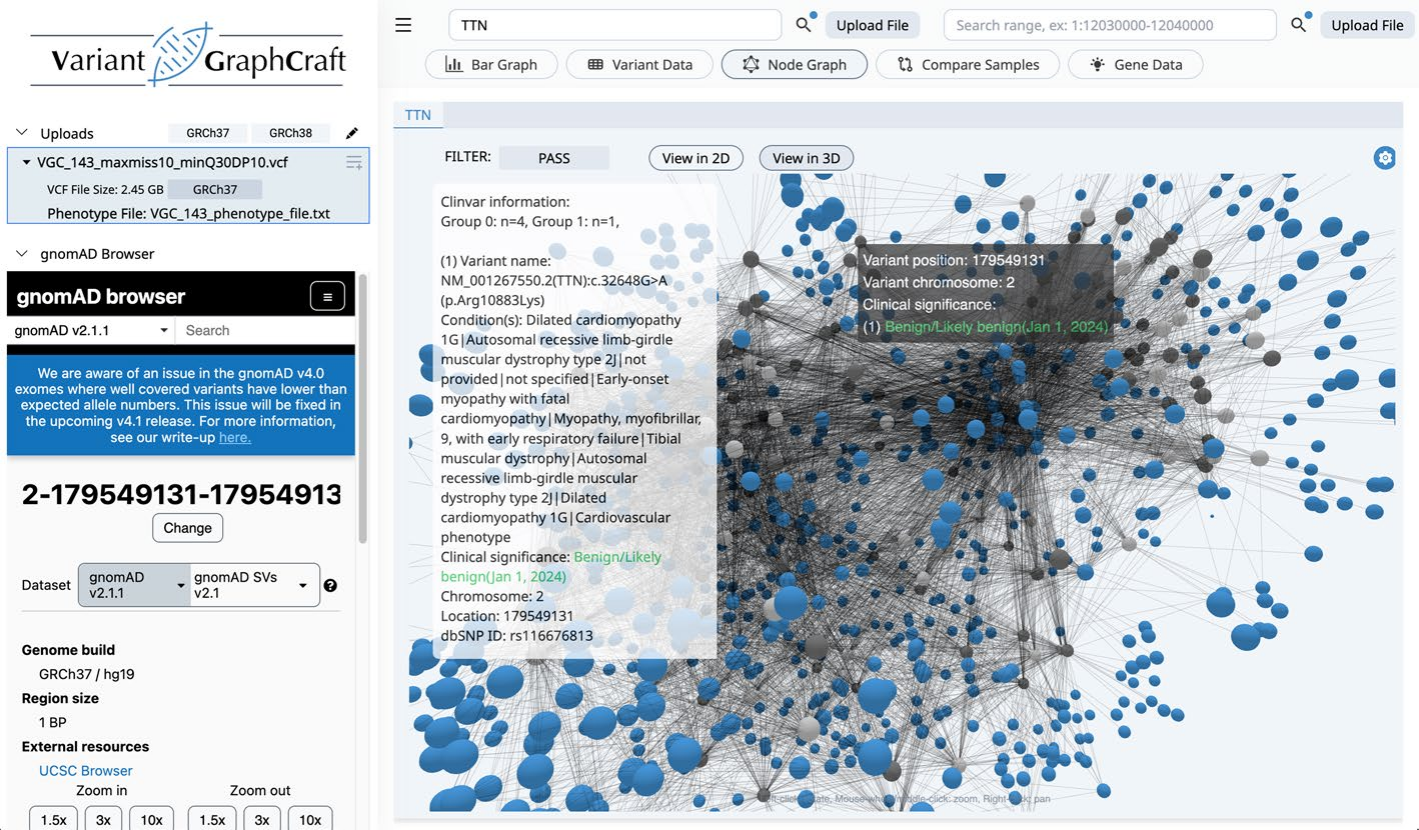
Force-graph visualization of variant to sample-grouping relations. Blue colored nodes show variants, while dark and light gray colored nodes represent cases and controls

VGC simplifies the interpretation of intricate genomic variation data by converting it into intuitive graphs, offering a visual summary of samples and their genotypes (Fig. 5). By representing genotype data graphically, VGC enables users to effortlessly recognize patterns of genetic variation across different samples. This graphical format aids in exploring the relationships between genotypes, making it easier to identify common variants or unique genetic patterns within a population. Such a visual method enriches the users' comprehension of the genetic landscape and assists in uncovering potential links between genotypes and phenotypes.

### Comparative analysis of VCF file analysis and visualization tools

To evaluate the effectiveness and unique features of VGC in comparison to other commonly used bioinformatics tools for VCF file analysis and visualization, we conducted a comprehensive comparison based on several criteria. These criteria include operating system compatibility, programming languages, user interfaces, Docker container support, genomic ranges support, variant annotation capabilities, interactive visualization features. We selected tools that have been published in peer-reviewed journals to ensure the reliability and scientific validation of the comparison. Table 1 provides a detailed comparison of VGC with tools such as VIVA, VCF-Server, BrowseVCF, VCFtools, IGV, VCF.Filter, GEMINI, and VCF-Miner. This table highlights the distinct advantages of VGC, such as dynamic filtering, interactive HTML5 visualization. The comparative analysis underscores VGC's strengths in providing a comprehensive, user-friendly, and efficient solution for VCF file analysis and visualization.

**Table 1:** Comparison of bioinformatics tools for VCF file analysis and visualization

Features	VIVA	VCF-Server	BrowseVCF	VCFtools	IGV	VCF.Filter	GEMINI	VCF-Miner	VGC
Standalone software	Yes	No	No	Yes	Yes	Yes	Yes	No	Yes
Operating System Compatibility	Cross-OS	Cross-OS	Cross-OS	Cross-OS	Cross-OS	Cross-OS	Cross-OS	Cross-OS	Cross-OS
Programming Language	Julia	C, Sails.js, Node.js	Python, JS, CSS, HTML5	Perl, C++	Java	Java	Python, SQLite	Java	Java, JavaScript
Interface	CLI, Jupyter	Web Service	GUI	CLI	GUI	GUI	CLI, GUI	GUI	GUI
Docker Container	Yes	Yes	No	No	Not listed	Not listed	No	No	No
Genomic Ranges Support	Yes	Not Specified	Yes	Yes	Not listed	Not listed	Yes	Yes	Yes
PASS Filter	Yes	Not Specified	Yes	Yes	Not listed	Not listed	Yes	Yes	Yes
Sample Selection	Yes	Yes	Yes	Yes	Not listed	Not listed	Yes	Yes	Yes
Variant Annotations	Yes	Yes	Yes	Yes	Not listed	Yes	Yes	Yes	Yes
Dynamic Filtering	Yes	Yes	Yes	Yes	Not listed	Not listed	Yes	Yes	Yes
Interactive HTML5 Visualization	Yes	Yes	No	No	Yes	No	No	No	Yes
Group Samples by Metadata Traits	Yes	Yes	Yes	Not Specified	Yes	Not listed	Yes	Not listed	Yes
Display Genotypic-Phenotypic Associations	Yes	Yes	Yes	Not Specified	Yes	Not listed	Yes	Not listed	Yes
Filtered Results as Tabular Data	Yes	Yes	Yes	Yes	Not listed	Not listed	Yes	Yes	Yes
Export Filtered VCF File	Yes	Yes	Yes	Yes	Yes	Not listed	Yes	Yes	No
Installation Required	Yes	No	Yes	Yes	Yes	Yes	Yes	Yes	Yes
Fully Open Source	Yes	Yes	Yes	Yes	Yes	Yes	Yes	Yes	Yes
Application Architecture	Standalone	Browser/Server	Browser/Server	Stand-alone	Stand-alone	Standalone	Standalone	Browser/Server	Standalone
GUI Engine	No GUI	HTML + Node.js	HTML + Python-CGI	Not listed	Java's Swing framework	Java's Swing framework	Not listed	Java's Swing framework	React, Tailwind CSS

## Discussion

The features of VGC provide a comprehensive solution for users to easily analyze and visualize genomic variation data in a fast and secure manner. One key advantage of the tool is its user-friendly interface, which allows users to easily navigate and analyze large datasets. Another noteworthy feature is the fast filtering of millions of variants, which is crucial for researchers dealing with large-scale genomic data. This feature ensures that users can quickly identify the most relevant variants for further analysis. After initial upload of VCF files, even large files can be visualized in seconds in the next sessions. The ability to add and query based on any number of user-defined groups (or phenotypes) is a significant advantage for researchers interested in studying specific groups of individuals or genes. This feature allows for more targeted analysis. The tool's ability to save and reuse analysis plans for reproducible research is a significant advantage, as it enables researchers to easily reproduce previous analyses and compare results. This feature is particularly important for ensuring that research findings are robust and reliable. The rapid VCF file browsing feature, with support for multiple visualizations such as histograms, spreadsheets, node graphs, and heatmaps, provides users with a comprehensive understanding of their data. This feature is particularly useful for identifying patterns and trends in genomic variation data. The tool’s ability to query by gene, range, position, and file upload, provides users with a range of options for searching and analyzing their data. This feature is particularly useful for identifying specific variants of interest and studying their potential impact on health and disease. The rapid identification and visualization of variant pathogenicity based on ClinVar data is another key advantage of VGC. This feature allows researchers to quickly identify potentially disease-causing variants, which can be further investigated for their clinical significance. VGC’s ability to display variant-to-sample genotype relations of user-defined groups is a significant advantage for researchers interested in studying the relationship between specific genetic variants and phenotypic traits. This feature allows for more targeted analysis and may lead to more insightful findings. The integrated variant querying through gnomAD, MSigDB, and Clinvar databases provides users with access to a wealth of public data, which can be used to enrich their own analysis. VGC supports both Human Genome Assembly Hg37 and GRCh38, significantly expanding its applicability and improving its accuracy by encompassing the most current genomic insights. This feature is particularly useful for identifying novel variants and potential disease-causing mutations. Finally, the software's design to run specifically on the local machine, with no VCF uploads to the cloud, ensures that users can work with their data in a secure and private environment. This feature is particularly important for researchers dealing with sensitive data and ensures that their research is conducted in a safe and confidential manner.

Despite these advancements, opportunities for further improvement remain. Integrating machine learning (ML) and large language models (LLMs) into VGC holds the promise of revolutionizing its capabilities in genomic analysis. Through predictive modeling, VGC could more effectively prioritize genetic variants of significance, while natural language processing (NLP) might automate the integration of scientific literature, enriching the context of variant data. Enhancing the tool's capacity to process even larger datasets would address existing scalability and efficiency challenges. Additionally, introducing more dynamic and customizable visualization options could further engage users by simplifying the interpretation of complex genomic data. A critical enhancement would be establishing a feedback system, enabling direct user input through GitHub or a dedicated site on Brown University's servers. This would allow the VGC team to quickly gather and act on user feedback, aligning the tool more closely with the genomic research community's evolving needs. Expanding integration with additional databases to capture emerging variant annotations and strengthening data privacy features, such as encrypted data storage, would also significantly enhance the tool's utility and user trust. Additionally, another potential future enhancement could involve implementing a feature that enables users to upload their own databases or annotation files. This functionality would allow users to annotate their VCF files using these personalized databases. By concentrating on these areas of development, VGC can continue to evolve to meet the growing demands of the genomic research community, offering state-of-the-art functionalities that keep pace with the latest developments in the field.

## Conclusions

In conclusion, the available features of VGC provide a comprehensive solution for researchers dealing with genomic variation data. The user-friendly interface, fast filtering, and ability to query based on user-defined groups, make it an efficient and effective tool for identifying potentially disease-causing variants. The ability to save and reuse analysis plans, rapid VCF file browsing, and integrated variant querying through public databases, further enhance the software’s capabilities, making it a valuable resource for genomic research. The tool’s rapid VCF file browsing with histogram, spreadsheet, node graph, and heatmap support further enhances its usability.

### Availability and requirements

Project name: Variant Graph Craft; Project home page: https://github.com/alperuzun/VGC; Operating system(s): Mac, Windows, Linux; Programming language: Java; Other requirements: Java 1.8 or higher; License: GPL-3.0 license. There no restrictions to use VGC by non-academics.

## Data Availability

All data generated or analyzed during this study are included in this published article (Schuster, J., et al. Protein Network Analysis of Whole Exome Sequencing of Severe Preeclampsia. *Front Genet* 2021;12:765985.).

## Abbreviations

VCF: Variant call format
VGC: Variant graph craft
HPO: Human Phenotype Ontology
MSigDB: Molecular Signatures Database
SNPs: Single nucleotide polymorphisms
IGV: Integrative genomics viewer
GEMINI: Genome Exploration and Mining INteractive Interface
VIVA: Visualization of variants
